# Amphipathic Helices Can Sense Both Positive and Negative Curvatures of Lipid Membranes

**DOI:** 10.1021/acs.jpclett.3c02785

**Published:** 2023-12-28

**Authors:** Peter Pajtinka, Robert Vácha

**Affiliations:** † CEITEC − Central European Institute of Technology, Masaryk University, Kamenice 753/5, 625 00 Brno, Czech Republic; ‡ National Centre for Biomolecular Research, Faculty of Science, Masaryk University, Kamenice 5, 625 00 Brno, Czech Republic; ¶ Department of Condensed Matter Physics, Faculty of Science, Masaryk University, Kotlářská 267/2, 611 37 Brno, Czech Republic

## Abstract

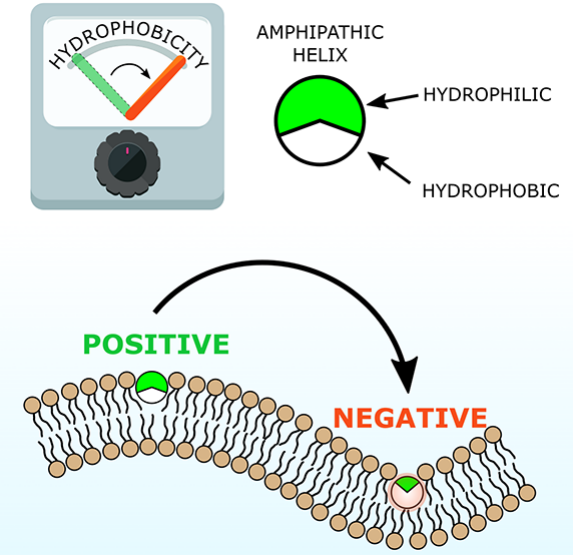

Curvature sensing is an essential ability of biomolecules to preferentially localize to membrane regions of a specific curvature. It has been shown that amphipathic helices (AHs), helical peptides with both hydrophilic and hydrophobic regions, could sense a positive membrane curvature. The origin of this AH sensing has been attributed to their ability to exploit lipid-packing defects that are enhanced in regions of positive curvature. In this study, we revisit an alternative framework where AHs act as sensors of local internal stress within the membrane, suggesting the possibility of an AH sensing a negative membrane curvature. Using molecular dynamics simulations, we gradually tuned the hydrophobicity of AHs, thereby adjusting their insertion depth so that the curvature preference of AHs is switched from positive to negative. This study suggests that highly hydrophobic AHs could preferentially localize proteins to regions of a negative membrane curvature.

The curvature of biological membranes is a tightly regulated biophysical property crucial for various cellular processes, including endocytosis, exocytosis, vesicle trafficking, and cellular signaling.^1,2^ To achieve such a level of regulation, cells have evolved specialized proteins and peptides that recognize and modulate membrane curvature.^3−9^ Membrane-associating amphipathic helices (AHs) belong to such curvature-recognizing molecules. These AHs are characterized by distinct regions of hydrophobic and polar residues and have been demonstrated to be potent sensors and inducers of membrane curvature.^1,10−13^ AHs are believed to sense positive membrane curvature due to their preference for lipid-packing defects. These defects are local membrane perturbations where lipid hydrophobic tails are exposed to an aqueous environment.^14^ Such defects are enhanced at positive membrane curvature^15^ due to the mismatch between the membrane curvature and the intrinsic curvature of lipids. These defects are then exploited and stabilized by the bulky hydrophobic residues of curvature-sensing helices.^14−16^

However, other studies employing continuum elastic theory and molecular dynamics (MD) simulations suggested that deeply inserted AHs might be able to sense a negative mean curvature.^17−19^ This possibility is not accounted for by the lipid-packing defects, and an alternative explanation is necessary. Campelo and Kozlov^18^ suggested that curvature-sensing helices can sense internal membrane stresses manifested on a molecular level as lipid-packing defects. Nevertheless, stress changes are not restricted to the region of lipid headgroups, and membrane curvature affects also lipid tails.^20,21^

In this work, we investigated whether AHs can be designed to sense a negative membrane curvature using label-free MD simulations, enabling us to study the specific effects of peptide sequences. For the membrane, we employed a buckled 1-palmitoyl-2-oleoyl-*sn*-glycero-3-phosphocholine (POPC) lipid bilayer (Figure 1A,B), which is a typical membrane model system that captures a range of membrane curvatures and has already been shown to be a suitable model for the study of the curvature-sensing ability of AHs^22−24^ (see extended methods in the Supporting Information for details). All simulations were performed with the GROMACS software package.^25^ Initially, we used the coarse-grained MARTINI force field (v2.2),^26−28^ which has repeatedly demonstrated its ability to describe lipid-packing defects accurately.^15^

**Figure 1:**
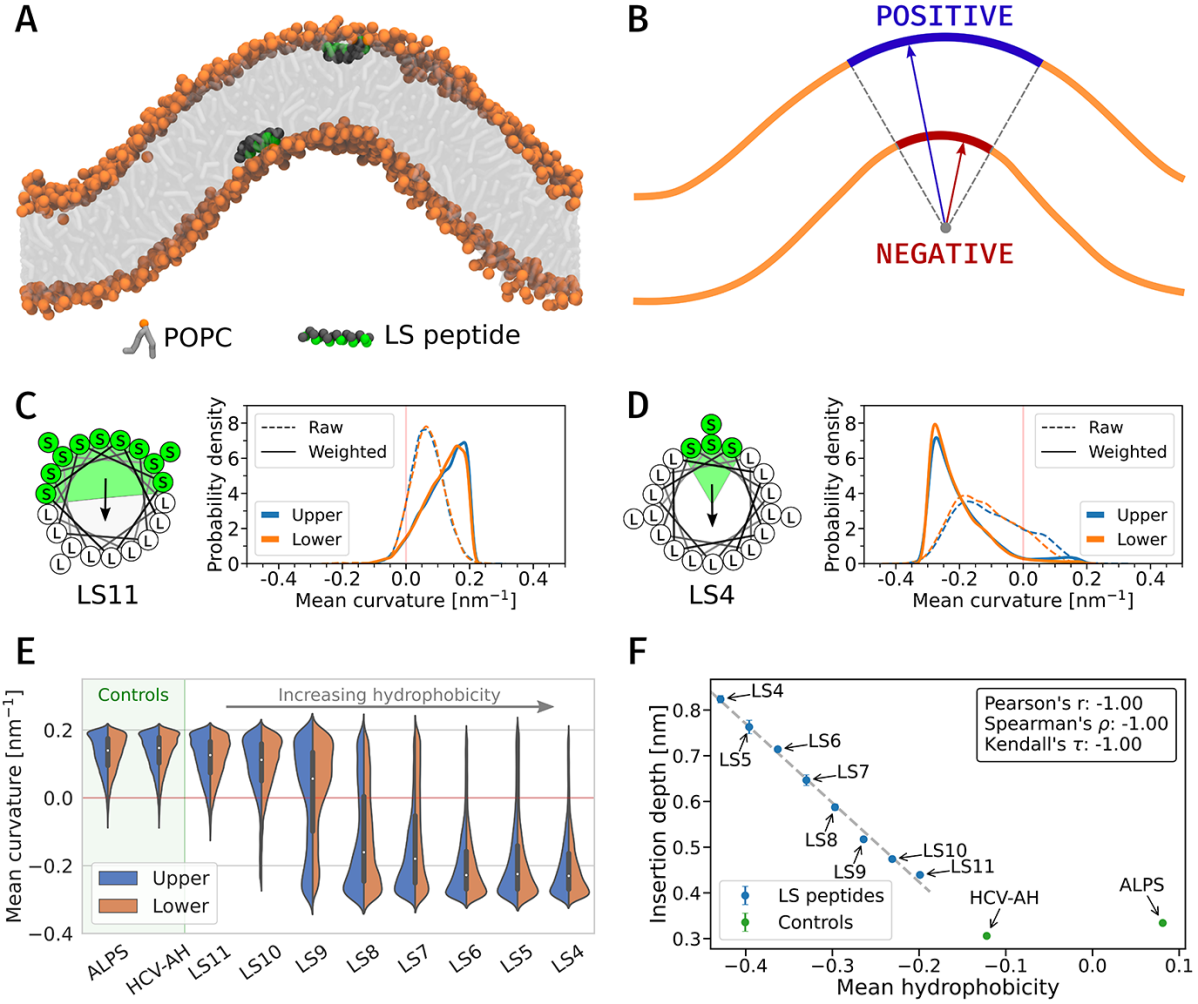
(A) Snapshot of the buckled POPC membrane used to evaluate the curvature-sensing ability of the studied peptides. In our simulations, we placed one peptide on each of the membrane leaflets. (B) Schematic diagram of analyzed membrane surfaces and curvatures present. (C and D) Helical wheel representation (left) and the membrane curvature sampled by the peptides (LS11 and LS4) throughout the simulation (right). Sampled curvature distributions of peptide on lower and upper leaflet is shown separately. (E) Weighted sampled mean membrane curvatures for the LS peptides, from the most hydrophilic (LS11) to the most hydrophobic (LS4), along with controls. Data are averaged over 3 independent replicas. Details about the weighting procedure are provided in the Supporting Information. (F) Insertion depth of studied peptides as a function of the mean hydrophobicity calculated using the Wimley–White hydrophobicity scale.^31^ Negative values indicate a favorable partitioning to a hydrophobic environment. The LS and control peptides are depicted as blue and green points, respectively.

We tested eight peptides gradually modified to tune their hydrophobicity and, consequently, their insertion depth into the lipid membrane, a factor previously demonstrated to significantly impact curvature generation^17^ and suggested to influence curvature sensing.^18,19^ For simplicity, amphipathic peptides containing only leucine and serine residues were considered, hereafter termed LS peptides. The peptides are labeled LS*X*, where *X* is the number of serine residues in the 21-residue peptide. In an α-helical conformation, the leucine and serine residues create continuous hydrophobic and hydrophilic patches, stabilizing the secondary structure after binding to the lipid membrane. The number of hydrophilic residues in the studied peptides ranged from half of the peptide to roughly 20% (see the helical wheels in Figure 1C,D). As a positive control, we included two known sensors of positive membrane curvature, namely, the amphipathic lipid-packing sensor motif from ArfGAP1 (ALPS)^3^ and the peptide derived from the NS5A protein of hepatitis C virus^29^ (HCV-AH). The sequences of all studied peptides are provided in the Supporting Information, Table S1. The membrane curvatures were analyzed using a fit of 2D surface to the membrane as in previous work by Bhaskara et al.^30^ based on the MemCurv scripts (https://github.com/bio-phys/MemCurv).

Our results demonstrate that the investigated peptides change their preference from the positive mean membrane curvature to negative as their hydrophobicity increases (see Figure 1E). The LS peptides with more than nine serine residues in their sequence, i.e., those peptides that are more hydrophilic, prefer the positive curvature. As the number of serine residues within the sequence decreased, the peptides became more hydrophobic, the propensity of the peptides to prefer negative membrane curvature increased. Subsequently, for LS peptides with less than nine serine residues, the average preferred mean curvature became negative. The most hydrophobic peptide we studied was LS4 peptide, which preferred a curvature as low as −0.23 nm^–1^ (median value, Figure 1D). As anticipated, the control peptides preferred membrane regions with positive mean curvature. The agreement of the results for peptides in the upper and lower leaflets starting from positions with different membrane curvature demonstrates the convergence of our results, and the differences from both leaflets could be used to estimate the sampling error.

The curvature preference of LS peptides is strongly correlated with the insertion depth of the peptides (Figure 1F). The shallowly adsorbed peptides preferred positive curvatures, while the most deeply inserted peptides favored negative curvatures. The increasing peptide hydrophobicity led to deeper peptide adsorption, and simultaneously, the peptides’ preference shifted toward negative mean membrane curvature (Figure 1E). The obtained linear dependence of insertion depth on the peptide’s mean hydrophobicity is likely due to the simple character of selected amino acids (leucine and serine). We anticipate more complex behavior for more diverse peptide sequences. Indeed, the correlation does not hold for control peptides (ALPS and HCV-AH) with inhomogeneous polar and apolar patches, which is in line with a previous report demonstrating that the chemistry and interactions of specific amino acids are important in peptide-generated membrane curvature.^32^

Note that the sampled mean curvature is affected by the distribution of accessible curvatures of the lipid bilayer. As seen from the schematic diagram in Figure 1B, regions of positive membrane curvature occupy a larger area than regions of negative curvature. This imbalance causes the appearance of bimodality in some of the distributions shown in Figure S2. To correct for the imbalance, we have analyzed the accessible curvature on the surface of the membrane buckle and used it to reweight the distributions of the sampled curvature, resulting in the distributions shown in Figure 1E. In other words, a peptide with no curvature preference would have sampled the curvature distribution equal to the accessible curvature, and the reweighted distribution would be uniform (for a more detailed discussion, see the Supporting Information). Nevertheless, even from the raw data presented in Figure S2, the gradual shift in preferred membrane curvature is evident, and the application of weighting only accentuates it even further (Figure 1E).

Note that the absolute numerical values of preferred curvature are not transferable, as it has been previously demonstrated for positive curvature sensing AHs that the theoretically preferred curvature lies outside the range of biologically accessible curvatures.^23^ Therefore, AHs will always prefer the largest curvature (the smallest radius) available, at least within the biologically relevant range of curvatures. We expect the same to hold for negative curvature sensing.

To verify the peptide preference for different curvatures obtained from coarse-grained simulations with the MARTINI 2 model, we performed additional simulations using the all-atom CHARMM36m force field.^33^ Due to the high computational demands of such simulations, we tested two peptides, LS11 and LS4, i.e., those with the most significant difference in their hydrophobicity/curvature preference, and ALPS as a control. We also performed simulations with the MARTINI 3 model^34^ to test the potential effect of the most recent coarse-grained parametrization. The control ALPS peptide favored positive curvature in all models (Figure 2A). For the LS4 peptide, there was an agreement in the negative curvature preference between MARTINI 2 and CHARMM36m simulations but not with the Martini 3 model. LS11 peptide favored the positive curvature in simulations with both coarse-grained MARTINI force fields. However, CHARMM36m simulations resulted in a very broad distribution of sampled curvature with the mean value at slightly negative curvature.

**Figure 2:**
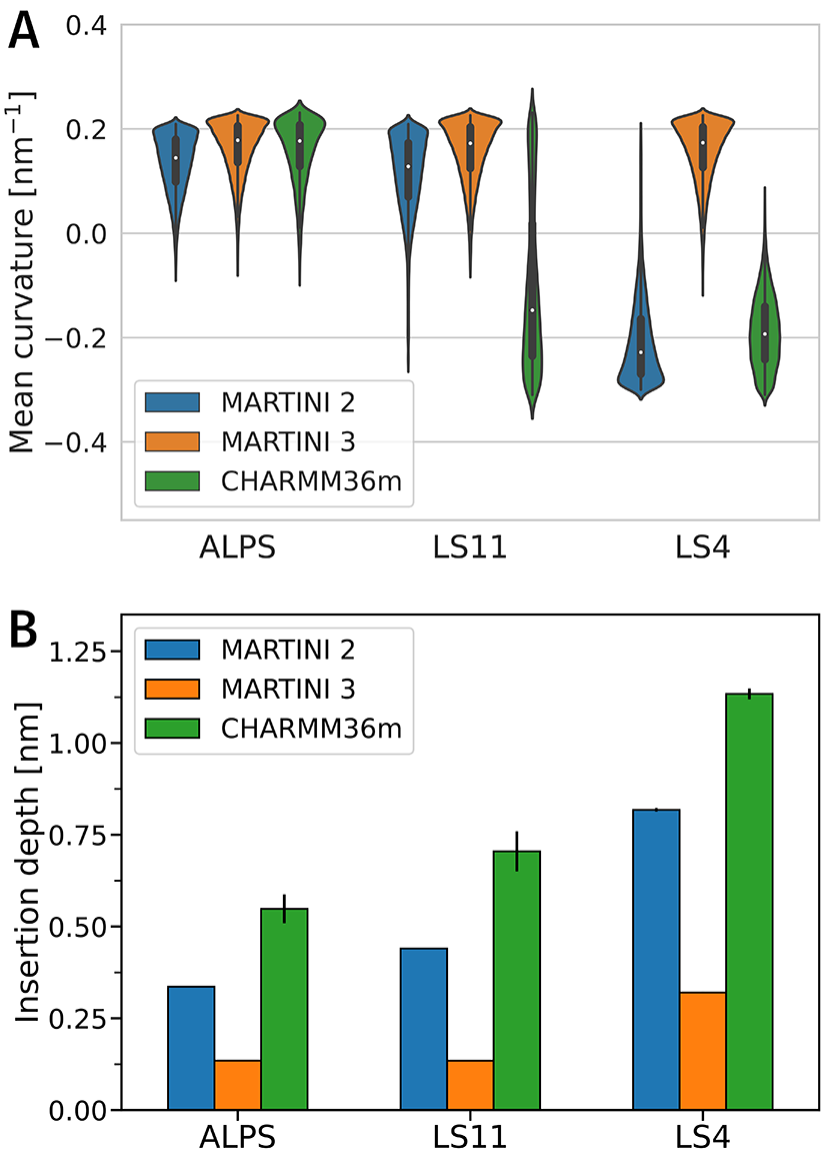
Comparison of results obtained with coarse-grained force fields MARTINI 2 and MARTINI 3, and all-atom CHARMM36m, for peptides ALPS, LS11, and LS4. (A) Weighted distributions of sampled mean membrane curvature. (B) Insertion depth of the peptides calculated as a distance between peptide’s center of mass and phosphate surface.

There was a consistent behavior that LS peptides adsorbed deeper with increasing hydrophobicity, following the decrease in the preferred mean curvature. MARTINI 3 was the least sensitive to changes in the peptide hydrophobicity. Even the most hydrophobic peptide, LS4, with only 4 serine residues and 17 leucine residues, inserted only very shallowly, which is unexpected for such a hydrophobic peptide. Results obtained with MARTINI2 agreed well with all-atom simulations, exhibiting the same trend with a roughly fixed offset: peptides inserted deeper using CHARMM36m. Following the correlation between the peptide’s preferred curvature and depth of insertion, deeper insertion in CHARMM36m simulations means that more hydrophilic peptides than LS6 would sense negative curvature in all-atom simulations. Indeed, LS11 in CHARMM36m simulations had a broad distribution of sampled curvatures with a slightly negative mean value (Figure 1F) similar to LS6 in simulations with MARTINI 2. However, the distribution was affected by a lower diffusion and subsequent slower convergence of all-atom simulations. In one replica, it took approximately 4 μs for a LS11 peptide to leave the region of positive membrane curvature to diffuse and remain in the region of negative curvature for the rest of the 6.5 μs long simulation (Figure S9). Overall, LS peptides behaved similarly in terms of depth insertion and curvature sensing in MARTINI 2 and CHARMM36m with an offset in which peptides inserted deeper and preferred more negative curvature in simulations with CHARMM36m.

Sensors capable of detecting negative membrane curvature, to the best of our knowledge, have been limited to large proteins such as I-BAR domains (inverted BAR).^35,36^ These proteins use their intrinsically curved surface to discriminate between different membrane curvatures. In contrast, AHs operate via a different mechanism. It is generally accepted that they sense lipid-packing defects. However, the lipid-packing defects are strongly suppressed in regions of negative curvature^14,15^ and, therefore, do not explain the preference for negative curvature observed here.

An alternative framework explains the curvature sensing of peripheral proteins through the sensing of internal membrane stresses.^18^ The sensing mechanism is based on the thermodynamic work required to form the cavity for the protein/helix insertion.^17,18^ This work is performed against the local internal membrane pressure, which is affected by membrane curvature. Indeed, the lipid tail order decreases in the negatively curved membranes, and this decrease is related to the increase of intramembrane stress (decrease of pressure).^20,21^ Therefore, a deeper insertion of protein helices is easier in negatively curved membranes, and helices that adsorb deeply into the membrane leaflet would prefer regions of the membrane with a negative curvature. This is in perfect agreement with our findings, demonstrating that the depth of peptide insertion (or adsorption) is one of the key determining factors in the curvature sensing. Thus, the internal membrane stress sensing model is a more general and applicable explanation/mechanism that also captures the negative curvature sensing of AHs.

The provided peptide examples and insights into curvature sensing of AHs could be applied to the optimization and design of peptides as antiviral agents^37,38^ and targeting of tumor-derived exosomes to support immunotherapy in cancer treatment.^39^ In addition, our findings may also be relevant for the sensing-related generation of membrane curvature, which has been proposed as one of the mechanisms for membrane pore formation by antimicrobial peptides^40^ and inhibition of viral fusion.^13^ It is worth noting that the absence of experimental evidence for negative membrane curvature sensing of AHs may be due to the high hydrophobicity of these peptides, which are experimentally challenging due to their low solubility and could be mislabeled as transmembrane domains.

In summary, we studied curvature sensing and the effect of the spontaneous insertion depth for a set of amphipathic helices composed of serine and leucine residues. Using coarse-grained and all-atom simulations with curved POPC bilayers, we identified peptides that are able to sense negative membrane curvature. In addition, we found a correlation between the peptide insertion depth in the membrane and the preferred mean curvature. Increasing the hydrophobicity of peptides resulted in deeper peptide insertion and a shift of its preferred mean curvature from positive to negative values. The provided first examples of peptides sensing negative curvature and their relation to the depth of insertion open a way for the design of new membrane sensors.

## Supporting Information Available

Simulation and analysis methods, list of used peptide sequences and performed simulations, helical wheel representation of the control peptides, raw distributions of sampled curvatures, secondary structure assessment from all-atom simulations, and time evolution of the sampled mean curvature for all-atom systems (PDF)
