# Supplementary material for: Amphipathic Helices Can Sense Both Positive and Negative Curvatures of Lipid Membranes
Source: J Phys Chem Lett. 2023 Dec 28;15(1):175–9. doi: 10.1021/acs.jpclett.3c02785 (PMC10788957; doi:10.1021/acs.jpclett.3c02785)
Supplement: Supplementary file 1 — jz3c02785_si_001.pdf [file jz3c02785_si_001.pdf]

Supporting Information

Amphiphatic Helices Can Sense Both Positive and Negative Curvatures of Lipid Membranes

Peter Pajtinka[†,‡] and Robert Vácha[*,†,‡,¶]

[†] CEITEC – Central European Institute of Technology, Masaryk University, Kamenice 753/5, 625 00 Brno, Czech Republic

[‡] National Centre for Biomolecular Research, Faculty of Science, Masaryk University, Kamenice 5, 625 00 Brno, Czech Republic

[¶] Department of Condensed Matter Physics, Faculty of Science, Masaryk University, Kotlářská 267/2, 611 37 Brno, Czech Republic

E-mail: robert.vacha@muni.cz

Methods

To investigate the effect of hydrophobicity on the membrane curvature sensing ability of peptides, we used coarse-grained (CG) and all-atom molecular dynamics simulations. All simulations were performed using GROMACS software package (versions 5.1.4 for MARTINI 2, 2021.2 and 2021.4 for MARTINI 3 and all-atom, respectively).[1] In our study, we used a buckled lipid bilayer, which has already been shown as a suitable model for the study of curvature sensing ability of amphipathic peptides,[2] trans-membrane proteins,[3] and lipid sorting.[4] The membrane buckle provides a variety of local curvatures as opposed to the almost uniform curvature distribution present in studies involving lipid vesicles.

First, the initially flat lipid bilayer comprising 1024 16:0-18:1 PC lipids (POPC) was generated using CHARMM-GUI Martini Maker[5,6] and underwent energy minimization using the steepest gradient method with the maximum force threshold of 100 $\mathrm{kJ\,mol^{-1}\,nm^{-1}}$. MARTINI v2.2 force field[7–9] with non-polarizable MARTINI water model was used at this point. Approximately 30 water beads per lipid were added together with ions, resulting in a 150 mM salt concentration. At that point, bilayer dimensions were approximately 25x13 $\mathrm{nm^2}$. The buckled membrane shape was achieved by compression of the membrane in the X direction. The bilayer plane dimensions after the compression were 21x13 $\mathrm{nm^2}$, corresponding to the value of compressional strain $\gamma \approx 0.16$.

The system preparation continued with a 200 ns long equilibration run, during which a temperature of 310 K was maintained using a stochastic velocity rescaling thermostat[10] with a coupling constant of 1 ps. Lipids and water with ions were coupled separately. To preserve the membrane's buckled shape, the system's compressibility was set to zero in X and Y directions. The pressure of 1 bar was kept constant using Berendsen barostat[11] with anisotropic coupling and coupling constant of 5 ps. Electrostatic interactions were treated using a reaction field with a relative dielectric constant of 15 and a cutoff radius of 1.1 nm. The same cutoff radius was applied to VdW interactions.

In the second step, the peptides were added. Peptides were constructed from the amino acid sequence as ideal alpha helices using Modeller:9v11 software.[12] Subsequently, the generated structures were coarse-grained and their helical structure was fixed. The choice of alpha helical secondary structure was validated by all-atom simulations (Figure S3).

One peptide copy per leaflet was then placed on the membrane buckle, slightly above the lipid

choline beads. After the peptide placement, the system was again equilibrated for 20 ns. The equilibration was followed by 20 μs long production run. For the production, Parinello-Rahman barostat[13,14] was used instead of Berendsen barostat with a coupling constant of 12 ps. All system coordinates were saved every 2 ns (1 ns for all-atom systems) for subsequent analysis. The first 5 μs were discarded from the analysis as additional equilibration. Three independent replicas were constructed for all MARTINI systems.

For MARTINI 3 systems, we used the final structures from the simulation of an empty buckled membrane simulated with MARTINI 2 parameters. The systems were simulated with MARTINI 3 (v3.0.0)[15] parameters for 20 μs, first 5 μs were discarded as equilibration. For the coarse-graining of the peptides generated from the sequence `martinize2.py`[16] was used. Apart from the aforementioned details, the simulation parameters were identical to MARTINI 2.

For all-atom simulations, similarly to MARTINI 3, two different snapshots from MARTINI 2 trajectories were selected and backmapped to all-atom using CHARMM-GUI All-atom converter,[17] which employs `backward.py`[18] to transform CG proteins into all-atom representation. As a result, we obtained systems of approximately half a million atoms. CHARMM36m[19] parameters were used to describe the system. Verlet integrator with a 2 fs integration step was employed. Electrostatic interactions were treated using PME[20] with a cut-off of 1.2 nm. The system's temperature was kept at 310 K using stochastic velocity rescaling thermostat[10] with separate coupling for solvent, membrane, and peptides. Semiisotropic pressure coupling was used with the Parinello-Rahman barostat[13,14] to maintain the pressure of 1 bar. Constraints were applied to bonds containing hydrogen atoms using the LINCS[21,22] algorithm. SETTLE[23] algorithm was used for water molecules. To preserve the buckled membrane shape, compressibility in the XY plane was set to zero as in CG systems, while the pressure in the Z-direction was allowed to fluctuate. The systems were simulated up to 6.5 μs. The first 1 μs was discarded as equilibration.

To analyze the membrane curvatures we employed approach used in previous work by Bhaskara et al.[3] based on the MemCurv scripts (https://github.com/bio-phys/MemCurv). The membrane shape from each trajectory frame was approximated by a 2D Fourier series, which was optimized using the least squares method with respect to the positions of phosphate beads or phosphorus atoms in the case of all-atom.

To precisely capture the local membrane shape, each membrane leaflet was fitted individually. Resulting differentiable surfaces were then used to calculate principal curvatures at the position of the peptide's center of mass (and corresponding mean and Gaussian curvatures).

The distance from bilayer midplane was calculated as a minimal distance between peptide backbone COM and the bilayer midplane as described by curved surface fitted to positions of innermost lipid tail beads. Similarly, the insertion depth was calculated as minimal distance between peptide backbone COM and the curved surface fitted to positions of phosphate beads or phosporus atoms, in the case of CG MARTINI and all-atom simulations, respectively.

Mean values and standard deviations were calculated from the average values for individual peptide copies in each replica and from independent replicas, three for CG systems and two for all-atom systems, resulting in overall n=6 and n=4 for CG and all-atom, respectively.

To predict the secondary structure of peptides from the all-atom trajectories, implementation of DSSP algorithm[24] (version 4.0.0) was used. Snapshots of the simulation systems were obtained using Visual Molecular Dynamics (VMD) software (version 1.9.3) and composed together in Inkscape (version 1.46.2). Analysis and plots were performed using Python 3, namely matplotlib (version 3.6.3), seaborn (version 0.12.2), and scipy (version 1.10.0) packages.

Analysis of accessible curvature and weighting

To obtain the accessible membrane curvature, we created a grid with equidistantly spaced points along the membrane buckle (X-direction). First, the trajectory of the membrane buckle was aligned according to the bilayer midplane (positions of C4A and C4B lipid beads). Subsequently, all frames were overlayed, and individual leaflets were fitted (positions of PO4 beads) using Fourier series (undulations in the Y direction are assumed to average out). We then selected points along the curve corresponding to the XZ profile of the membrane, dividing the curve into intervals of equal arc length. The points along the Y direction were generated equidistantly. The resulting grid had 102 points in X and 62 in Y direction. Mean membrane curvature was then calculated at each grid node for every 100th frame of the simulation trajectory, providing us with the distribution of accessible curvature.

To filter the effect of accessible curvature, we used the distribution of curvature present on the membrane surface (surface given by fitting PO4 beads of upper or lower leaflet) and inverted it to give higher weight to the less represented curvature values. To avoid overemphasizing the curvatures that arose only transiently, the curvature values visited less than in 0.1% of the frames were discarded from the reweighing. Some peptides have ability to generate and stabilize certain curvatures, affecting the overall distribution. However, in our systems, there concentration of peptides was low and the same applied for the difference between the respective distributions. A common reference distribution was therefore used, in our case LS4.

Table S1: Summary of peptide labels used in this work, together with corresponding amino acid sequences, mean hydrophobicity according to the Wimley-White hydrophobicity scale,[25] and mean hydrophobic moment, $\langle \mu \rangle$.

Peptide	Sequence	Length	Hydrophobicity	$\langle \mu \rangle$
ALPS (ArfGAP1)	`DDFLNSAMSSLYSGWSSFTTGASKFASAAKEGATK`	36	0.081	0.269
HCV-AH	`SGSWLRDVWDWICTVLTDFKTWLQSKL`	27	-0.122	0.285
LS11	`LSSLLSLLSSLLSSLSSLLSS`	21	-0.199	0.218
LS10	`LSSLLSLLSSLLSLLSSLLSS`	21	-0.231	0.212
LS9	`LSSLLSLLSSLLSLLSSLLSL`	21	-0.264	0.200
LS8	`LSLLLSLLSSLLSLLSSLLSL`	21	-0.297	0.192
LS7	`LSLLLSLLSLLLSLLSSLLSL`	21	-0.330	0.179
LS6	`LSLLLSLLSLLLSLLSLLLSL`	21	-0.363	0.160
LS5	`LSLLLLLLSLLLSLLSLLLSL`	21	-0.396	0.137
LS4	`LSLLLLLLSLLLSLLLLLLSL`	21	-0.429	0.110

Table S2: Summary of performed simulations, specifying if the simulation was run using MARTINI 2 (M2), MARTINI 3 (M3), or all-atom CHARMM36m (AA). The number of lipid molecules and the overall size of the system (beads or atoms) along with the simulation time are listed as well.

Peptide	AA/CG	Replicas	N_{lipids}	System size	Simulation time [μs]
ALPS	M2	3	1024	44234	20/20/20
HCV-AH	M2	3	1024	44229	20/20/20
LS11	M2	3	1024	44194	20/20/20
LS10	M2	3	1024	44190	20/20/20
LS9	M2	3	1024	44180	20/20/20
LS8	M2	3	1024	44185	20/20/20
LS7	M2	3	1024	44187	30/20/20
LS6	M2	3	1024	44183	30/20/20
LS5	M2	3	1024	44175	20/20/20
LS4	M2	3	1024	44181	25/20/20
ALPS	M3	3	1024	43755	20/20/20
LS11	M3	3	1024	43716	20/20/20
LS4	M3	3	1024	43717	20/20/20
ALPS	AA	2	1024	509064	2/2
LS11	AA	2	1028	509554	4.8/6.5
LS4	AA	2	1028	509510	4.8/5

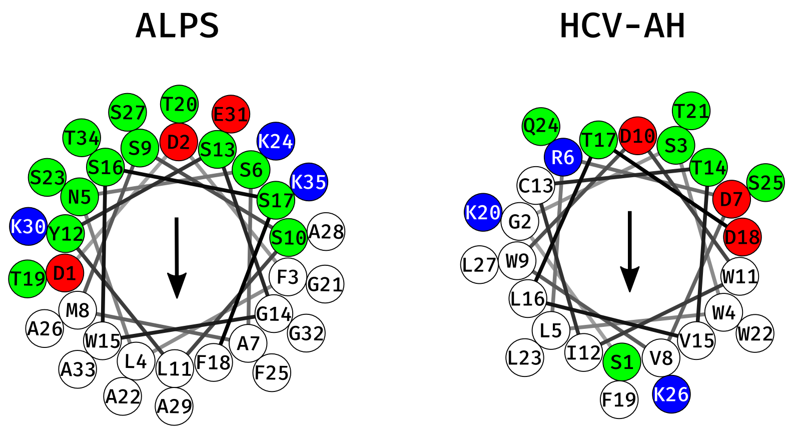

Figure S1: Helical wheel representations of control peptides, amphipathic lipid packing sensor of ArfGAP1 (ALPS) and amphipathic helix derived from the nonstructural protein NS5A of hepatitis C virus. Residues are colored according to their properties: acidic (red), basic (blue), nonpolar (white), and polar (green).

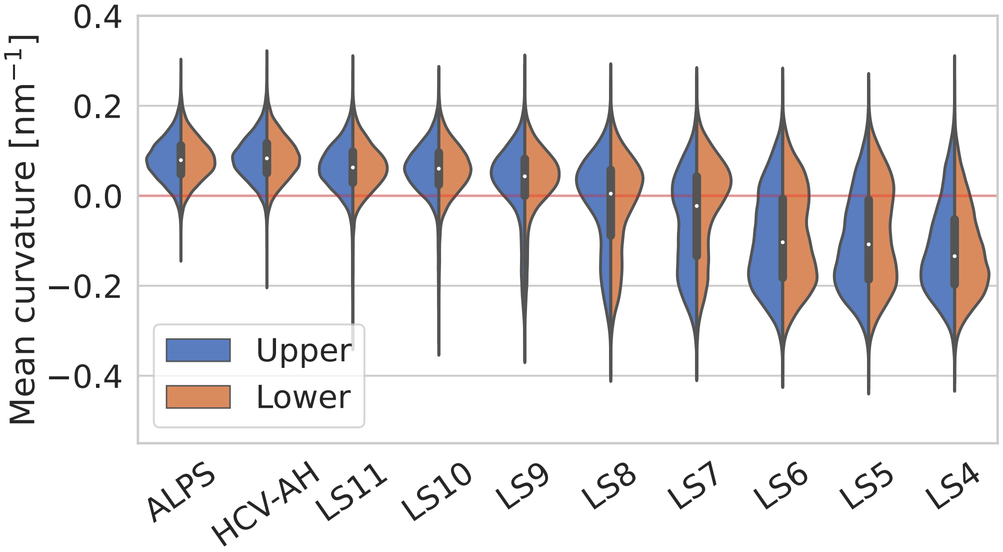

Figure S2: Summary of distributions of sampled mean membrane curvatures by different peptides in MARTINI 2. Data for peptide copies in individual leaflets are plotted separately. The red line serves as a guide for the eye and shows the position of zero mean curvature.

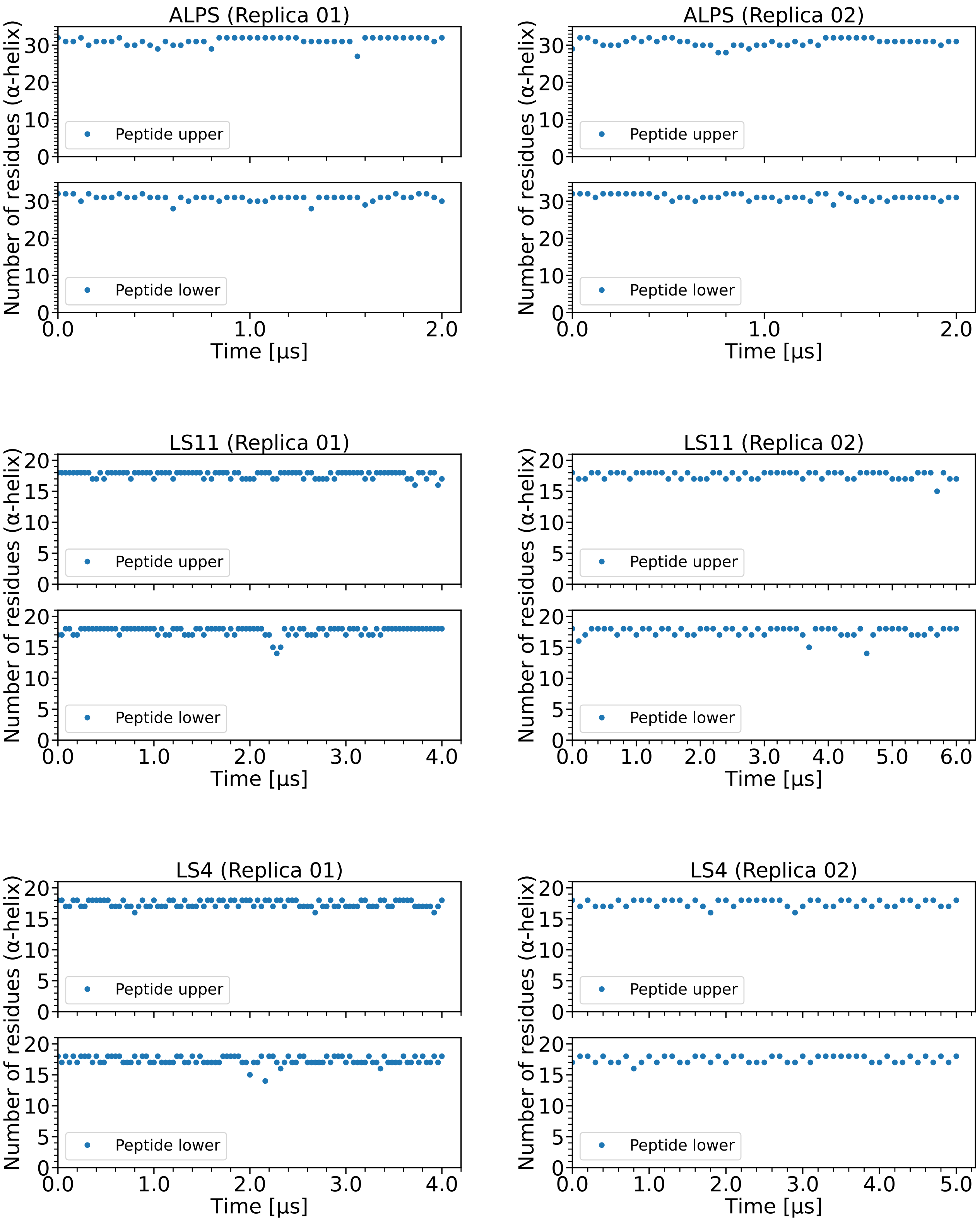

Figure S3: Time evolution of α-helical propensity in all-atom simulations of ALPS, LS11, and LS4, evaluated using DSSP algorithm.[24] Data for copies of peptides on both membrane leaflets and independent replicas are shown separately. Trajectory frames were analyzed every 100 ns.

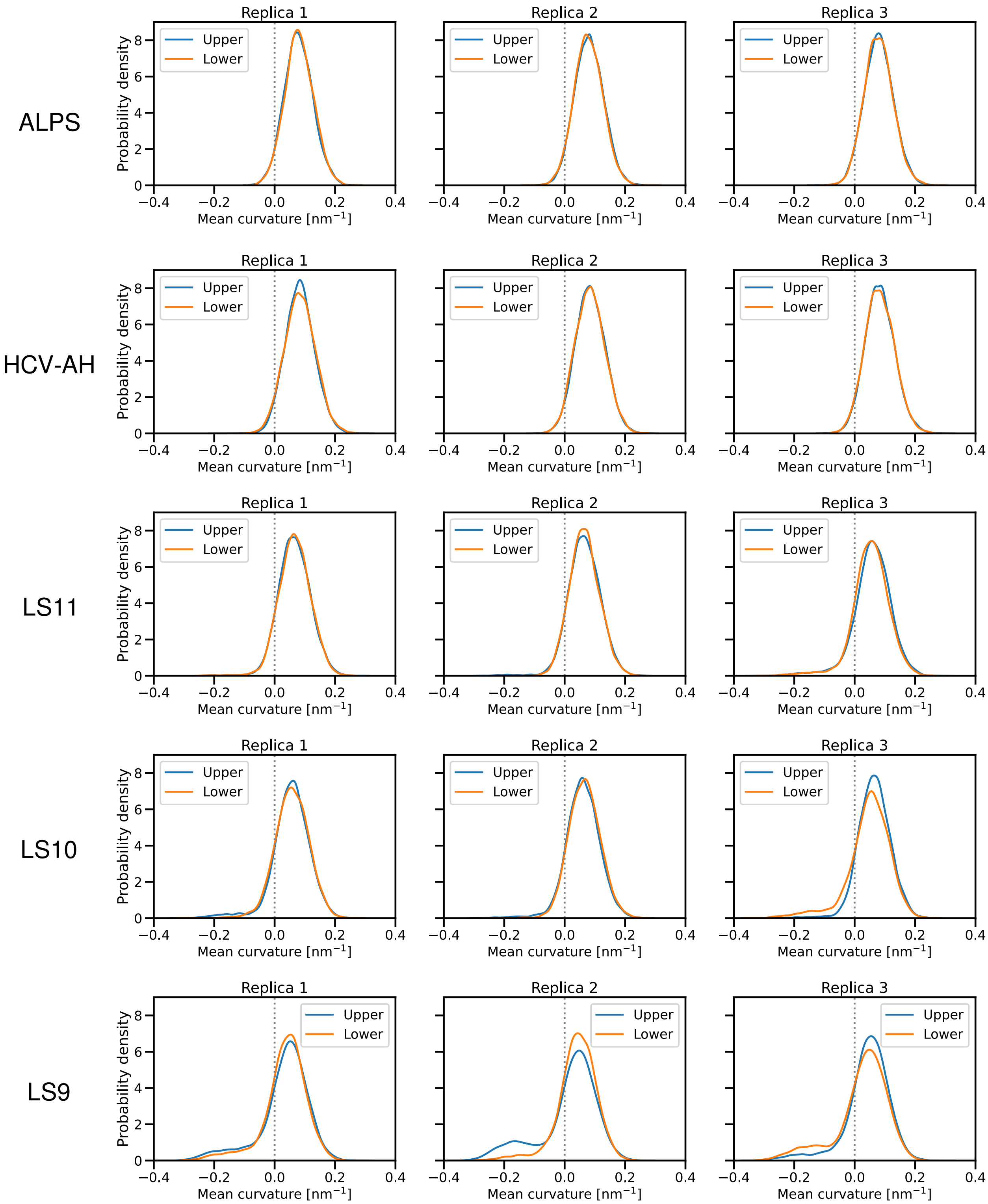

Figure S4: Distributions of sampled mean membrane curvatures by different peptides in MARTINI 2 shown for individual replicas. Data for peptide copies in individual leaflets are plotted separately. The dotted grey line serves as a guide for the eye and shows the position of zero mean curvature.

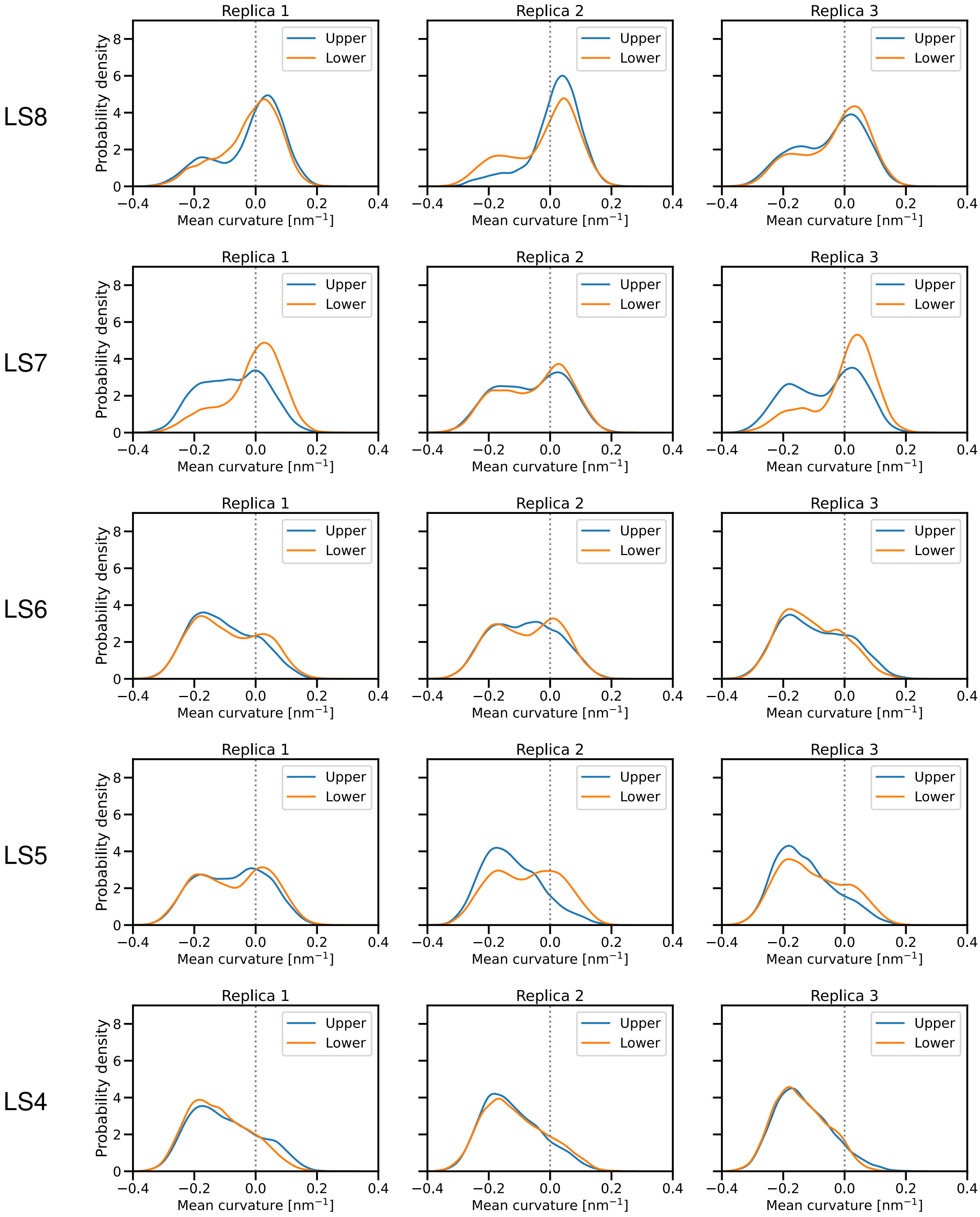

Figure S5: Distributions of sampled mean membrane curvatures by different peptides in MARTINI 2 shown for individual replicas. Data for peptide copies in individual leaflets are plotted separately. The dotted grey line serves as a guide for the eye and shows the position of zero mean curvature.

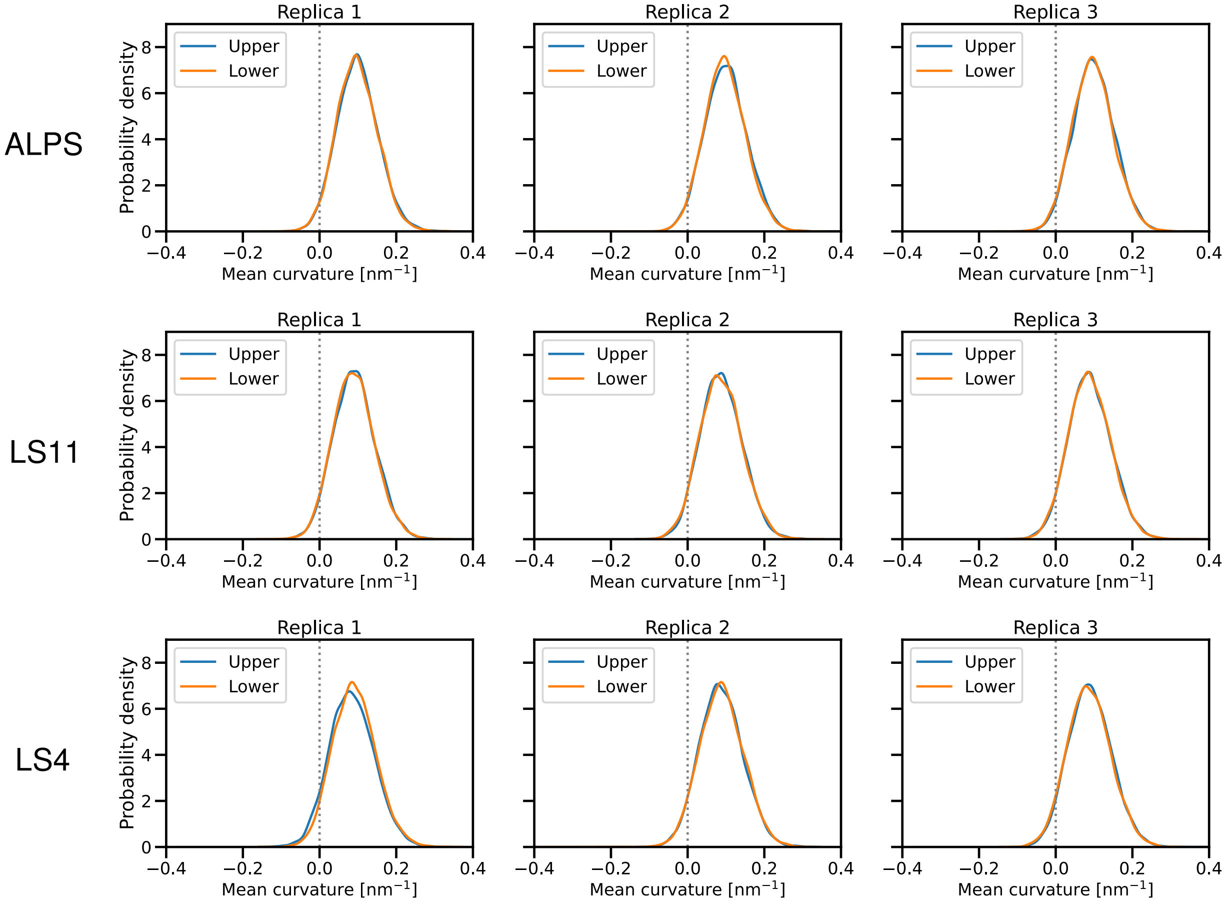

Figure S6: Distributions of sampled mean membrane curvatures by different peptides in MARTINI 3 shown for individual replicas. Data for peptide copies in individual leaflets are plotted separately. The dotted grey line serves as a guide for the eye and shows the position of zero mean curvature.

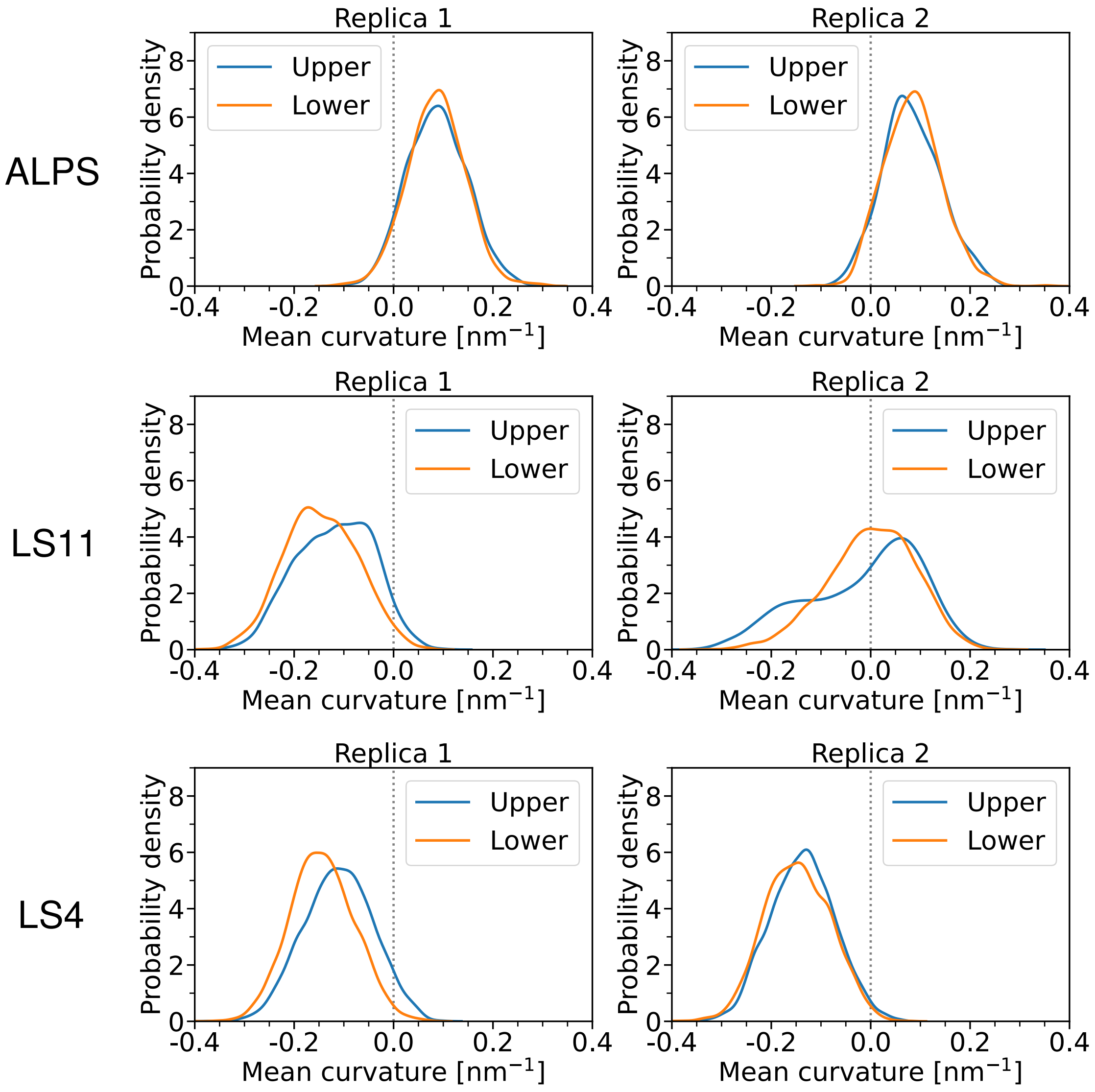

Figure S7: Distributions of sampled mean membrane curvatures by different peptides in all-atom CHARMM36m. Data for peptide copies in individual leaflets are shown. The dotted grey line serves as a guide for the eye and shows the position of zero mean curvature.

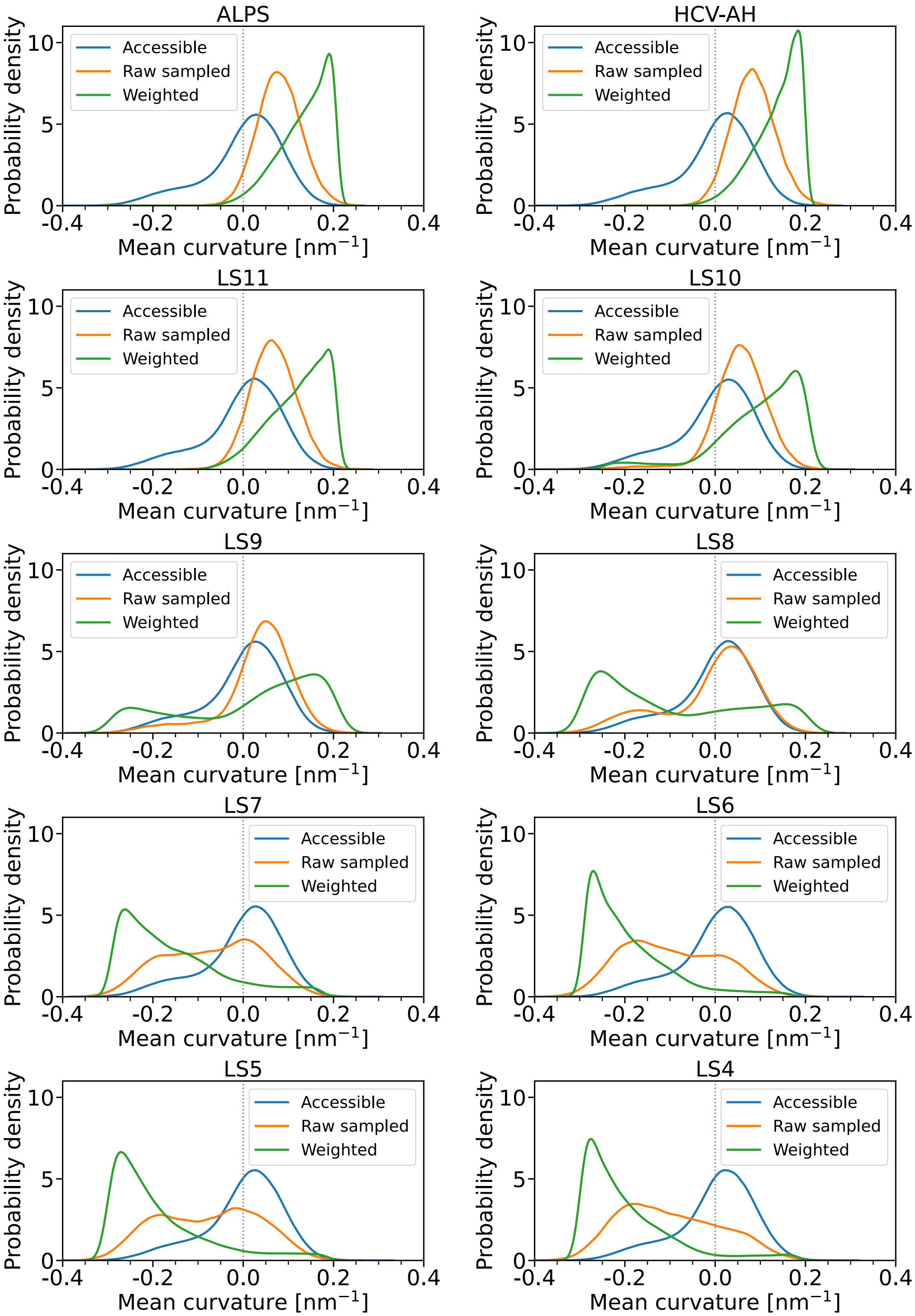

Figure S8: Plots showing the distributions of reweighted/normalized mean membrane curvatures by different peptides, together with raw sampled data and distribution of accessible curvature on membrane surface (phosphate beads) in coarse-grained MARTINI 2 force field. Data for peptide copies in individual leaflets are shown. The dotted grey line serves as a guide for the eye and shows the position of zero mean curvature.

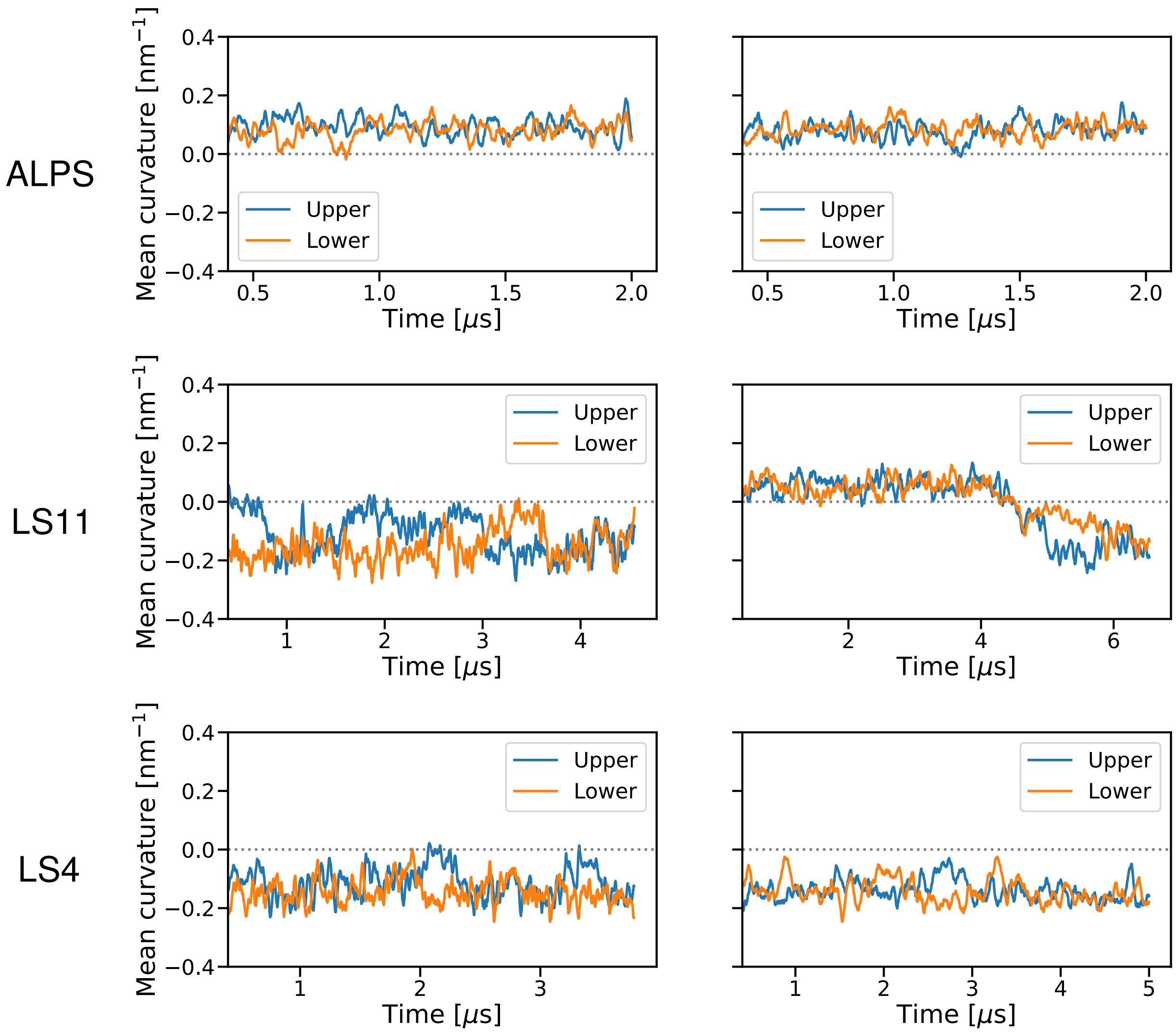

Figure S9: Time evolution of the membrane mean curvature sampled by the individual peptides in all-atom representation on buckled membrane shown as a 200 ns moving average. Data from both replicas are shown.

References

(1) Abraham, M. J.; Murtola, T.; Schulz, R.; Páll, S.; Smith, J. C.; Hess, B.; Lindahl, E. GROMACS: High performance molecular simulations through multi-level parallelism from laptops to supercomputers. *SoftwareX* **2015**, *1*, 19–25.

(2) Gómez-Llobregat, J.; Elías-Wolff, F.; Lindén, M. Anisotropic membrane curvature sensing by amphipathic peptides. *Biophys. J.* **2016**, *110*, 197–204.

(3) Bhaskara, R. M.; Grumati, P.; Garcia-Pardo, J.; Kalayil, S.; Covarrubias-Pinto, A.; Chen, W.; Kudryashev, M.; Dikic, I.; Hummer, G. Curvature induction and membrane remodeling by FAM134B reticulon homology domain assist selective ER-phagy. *Nat. Commun.* **2019**, *10*, 1–13.

(4) Boyd, K. J.; Alder, N. N.; May, E. R. Buckling under pressure: curvature-based lipid segregation and stability modulation in cardiolipin-containing bilayers. *Langmuir* **2017**, *33*, 6937–6946.

(5) Lee, J.; Cheng, X.; Swails, J. M.; Yeom, M. S.; Eastman, P. K.; Lemkul, J. A.; Wei, S.; Buckner, J.; Jeong, J. C.; Qi, Y., et al. CHARMM-GUI input generator for NAMD, GROMACS, AMBER, OpenMM, and CHARMM/OpenMM simulations using the CHARMM36 additive force field. *J. Chem. Theory Comput.* **2016**, *12*, 405–413.

(6) Qi, Y.; Ingólfsson, H. I.; Cheng, X.; Lee, J.; Marrink, S. J.; Im, W. CHARMM-GUI Martini maker for coarse-grained simulations with the Martini force field. *J. Chem. Theory Comput.* **2015**, *11*, 4486–4494.

(7) Marrink, S. J.; Risselada, H. J.; Yefimov, S.; Tieleman, D. P.; De Vries, A. H. The MARTINI force field: coarse grained model for biomolecular simulations. *J. Phys. Chem. B* **2007**, *111*, 7812–7824.

(8) Monticelli, L.; Kandasamy, S. K.; Periole, X.; Larson, R. G.; Tieleman, D. P.; Marrink, S.-J. The MARTINI coarse-grained force field: extension to proteins. *J. Chem. Theory Comput.* **2008**, *4*, 819–834.

(9) De Jong, D. H.; Singh, G.; Bennett, W. D.; Arnarez, C.; Wassenaar, T. A.; Schafer, L. V.; Periole, X.; Tieleman, D. P.; Marrink, S. J. Improved parameters for the martini coarse-grained protein force field. *J. Chem. Theory Comput.* **2013**, *9*, 687–697.

(10) Bussi, G.; Donadio, D.; Parrinello, M. Canonical sampling through velocity rescaling. *J. Chem. Phys.* **2007**, *126*, 014101.

(11) Berendsen, H. J.; Postma, J. v.; van Gunsteren, W. F.; DiNola, A.; Haak, J. R. Molecular dynamics with coupling to an external bath. *J. Chem. Phys.* **1984**, *81*, 3684–3690.

(12) Webb, B.; Sali, A. Comparative protein structure modeling using MODELLER. *Curr. Protoc. Bioinform.* **2016**, *54*, 5–6.

(13) Parrinello, M.; Rahman, A. Crystal structure and pair potentials: A molecular-dynamics study. *Phys. Rev. Lett.* **1980**, *45*, 1196.

(14) Parrinello, M.; Rahman, A. Polymorphic transitions in single crystals: A new molecular dynamics method. *J. Appl. Phys.* **1981**, *52*, 7182–7190.

(15) Souza, P. C. T. et al. Martini 3: a general purpose force field for coarse-grained molecular dynamics. *Nat. Methods* **2021**, *18*, 382–388.

(16) Kroon, P. C.; Grunewald, F.; Barnoud, J.; van Tilburg, M.; Souza, P. C. T.; Wassenaar, T. A.; Marrink, S. J. Martinize2 and Vermouth: Unified Framework for Topology Generation. **2023**, DOI: `10.7554/elife.90627.1`.

(17) Jo, S.; Kim, T.; Iyer, V. G.; Im, W. CHARMM-GUI: a web-based graphical user interface for CHARMM. *J. Comput. Chem.* **2008**, *29*, 1859–1865.

(18) Wassenaar, T. A.; Pluhackova, K.; Böckmann, R. A.; Marrink, S. J.; Tieleman, D. P. Going backward: a flexible geometric approach to reverse transformation from coarse grained to atomistic models. *J. Chem. Theory Comput.* **2014**, *10*, 676–690.

(19) Huang, J.; Rauscher, S.; Nawrocki, G.; Ran, T.; Feig, M.; De Groot, B. L.; Grubmüller, H.; MacKerell Jr, A. D. CHARMM36m: an improved force field for folded and intrinsically disordered proteins. *Nat. Methods* **2017**, *14*, 71–73.

(20) Essmann, U.; Perera, L.; Berkowitz, M. L.; Darden, T.; Lee, H.; Pedersen, L. G. A smooth particle mesh Ewald method. *J. Chem. Phys.* **1995**, *103*, 8577–8593.

(21) Hess, B.; Bekker, H.; Berendsen, H. J.; Fraaije, J. G. LINCS: A linear constraint solver for molecular simulations. *J. Comput. Chem.* **1997**, *18*, 1463–1472.

(22) Hess, B. P-LINCS: A parallel linear constraint solver for molecular simulation. *J. Chem. Theory Comput.* **2008**, *4*, 116–122.

(23) Miyamoto, S.; Kollman, P. A. Settle: An analytical version of the SHAKE and RATTLE algorithm for rigid water models. *J. Comput. Chem.* **1992**, *13*, 952–962.

(24) Kabsch, W.; Sander, C. Dictionary of protein secondary structure: pattern recognition of hydrogen-bonded and geometrical features. *Biopolymers* **1983**, *22*, 2577–2637.

(25) Wimley, W. C.; White, S. H. Experimentally determined hydrophobicity scale for proteins at membrane interfaces. *Nat. Struct. Biol.* **1996**, *3*, 842–848.
